# Co-Precipitation of YAG Powders for Transparent Materials: Effect of the Synthesis Parameters on Processing and Microstructure

**DOI:** 10.3390/ma7107145

**Published:** 2014-10-22

**Authors:** Paola Palmero, Rebecca Traverso

**Affiliations:** Department of Applied Science and Technology, Politecnico di Torino, INSTM R.U. PoliTO, Laboratorio di Tecnologia ed Ingegnerizzazione dei Materiali Ceramici (LINCE), Corso Duca degli Abruzzi, 24, Torino 10129, Italy; E-Mail: rebecca.traverso@polito.it

**Keywords:** YAG, co-precipitation, precipitant, phase evolution, sintering, microstructure

## Abstract

The fabrication of transparent polycrystalline Y_3_Al_5_O_12_ (YAG) is still a challenge, requiring the achievement of highly pure and fully dense, homogeneous materials. An important role is played by the powder characteristics: pure, fine and unagglomerated powders are essential for achieving full density and the required microstructural features. Keeping in mind these requirements, the aim of this work was to investigate the role of different synthesis parameters during co-precipitation, which is widely used to prepare YAG powders for transparent devices. The key role of the precipitant solution is here illustrated, by comparing the effect of aqueous ammonia (AA) *versus* ammonium hydrogen carbonate (AHC). This latter allowed the preparation of softly agglomerated powders, characterized by a very good sinterability. However, when AHC is used, attention should be paid to its concentration. By comparing the effect of two AHC precipitant solutions (at 0.5 M and 1.5 M, respectively), only the former one allowed the preparation of pure YAG powders, free from secondary phases. In this last condition, by using both chlorides and nitrates as YAG precursors, pressureless sintering at 1500–1600 °C/3 h gave rise to pure materials, highly dense and characterized by a fine and homogeneous microstructure.

## 1. Introduction

Fully dense polycrystalline Y_3_Al_5_O_12_ (YAG) can be used as a structural material, in particular for high-temperature applications, due to the high melting point (about 1940 °C [1]), resistance to corrosive conditions [2,3] and low creep rate [4].

In addition, due to its cubic structure, YAG is suitable for transparent devices. In 1995, Ikesue *et al.* [5] first fabricated a transparent polycrystalline YAG material (doped with Nd) for laser applications. This material, with nearly the same optical characteristics as those of single crystal, was prepared by solid-state reaction method, reaching 99.98% density and grain size of about 50 μm.

Techniques for producing polycrystalline transparent YAG have evolved ever since. In fact, unlike other transparent ceramics such as alumina, YAG is a monorefringent material due to the cubic structure. This means that the grain size is not crucial in terms of optical transmittance, but is still important with respect to the mechanical properties [6]. Consequently, efforts have concentrated on the production of fine-grained YAG, able to provide good mechanical properties in addition to transparency [6,7].

For this reason, besides the use of increasingly advanced sintering techniques [6,7,8,9], attention is paid to the synthesis routes, since the powder characteristics can affect in a remarkable way the final product. Fine, unagglomerated powders, with narrow size distribution are essential for achieving full density and the required microstructural features. In addition, powders should exhibit proper chemical and phase purity, to avoid loss of transparency due to scattering phenomena by secondary phases [10]. In conventional solid-state reaction between the constituent oxides, a high calcination temperature (higher that 1600 °C), carried out for a prolonged time, is necessary to gain the pure YAG phase, resulting in significant grain growth [11] and poor mechanical properties. In addition, the extensive milling of the oxide powders leads to possible contamination, thus decreasing the optical properties [10]. For this reason, several wet chemical methods have been developed and used for the low-temperature preparation of pure YAG powders. These methods include sol-gel processing [12], hydrothermal synthesis [13], glycol-thermal method [14] and co-precipitation [15,16]. In these chemical processes, the reactant cations achieve intimate mixing on the atomic level, leading to an increase in phase purity and a decrease in synthesis temperature. In spite of this, the formation of secondary phases during YAG preparation by wet chemical routes is still an issue [17]. In addition, the ultrafine powders prepared by these methods present the major drawback of a severe agglomeration [15], causing poor sinterability.

In this paper, we prepared YAG powders by a co-precipitation route, by comparing and optimizing different synthesis parameters. First, we compared the effect of two different precipitants, aqueous ammonia (AA) and ammonium hydrogen carbonate (AHC). In this latter case, we investigated the role of the precipitant concentration, by using AHC at 0.5 M and 1.5 M. Finally, once the best precipitation conditions were fixed, the role of the precursors (aluminum and yttrium chlorides *versus* nitrates) was investigated.

The green bodies were prepared by slip casting, since this technique is able to produce homogeneous and close-packed green microstructure [18]. In spite of this, slip casting is rarely investigated as a YAG forming method, in comparison with other routes such as dry-pressing [11] and isostatic pressing [5,15].

In this work, we investigated the role of the previously mentioned synthesis parameters on the phase evolution, dispersability, sinterability and final microstructures, with the aim of preparing pure YAG powders suitable for the fabrication of transparent materials.

## 2. Experimental Section

### 2.1. Materials

The yttrium and aluminum sources for the YAG syntheses were both chlorides and nitrates. Namely, YCl_3_·6H_2_O (99.99% purity), AlCl_3_·9H_2_O (>99.0% purity), Y(NO_3_)_3_·6H_2_O (99.8% purity) and Al(NO_3_)_3_·9H_2_O (>98.5% purity), all supplied by Sigma-Aldrich, were used. The aqueous solutions containing Al and Y chlorides or nitrates (molar ratio 5:3) were prepared at 0.32 M.

Two precipitant solutions were used: dilute aqueous ammonia (hereafter referred to as AA), and ammonium hydrogen carbonate (referred to as AHC). The latter was employed at two molar concentrations: 0.5 M and 1.5 M. In all the preparations, for each litre of salts solution, 3 L of the precipitant solution were employed.

### 2.2. Syntheses by Co-Precipitation Route

The powder synthesis was carried out by reverse-strike co-precipitation. Chloride precursors were firstly used.

When AA was employed, the mixed salts solution was added dropwise to the precipitant solution. The pH was kept constant at the value of 9 (to assure the complete and simultaneous precipitation of both yttrium and aluminum hydroxides) by addition of extra-ammonia solution. The pH was continuously monitored by a pH-meter, to allow only very small pH fluctuations (±0.2 pH units).

When AHC was employed, no extra-precipitant was added during the syntheses. Therefore, during precipitation, the pH progressively decreased from the starting value of about 8.5–9 to about 7–6.5. Two syntheses were carried out, differing only on the AHC concentration (0.5 or 1.5 M).

In the case of AHC at 0.5 M, a further co-precipitation was carried out, by using yttrium and aluminum nitrates as YAG precursors.

In all the cases, the gelatinous precipitates were washed by centrifugation several times, first in distilled water (four times) and then in absolute ethanol (twice). Finally, the gelatinous products were dried in an oven, at 60 °C. For sake of clarity, Table 1 collects the different syntheses conditions and the respective material designations.

**Table 1:** Materials designation as a functional of the co-precipitation experimental conditions.

Designation	Precursors	Precipitant
YAG_cl_-AA	YCl_3_·6H_2_O and AlCl_3_·9H_2_O	Ammonium hydroxide solution, dilute
YAG_cl_-AHC0.5	YCl_3_·6H_2_O and AlCl_3_·9H_2_O	Ammonium hydrogen carbonate 0.5 M
YAG_cl_-AHC1.5	YCl_3_·6H_2_O and AlCl_3_·9H_2_O	Ammonium hydrogen carbonate 1.5 M
YAG_n_-AHC0.5	Y(NO_3_)_3_·6H_2_O and Al(NO_3_)_3_·9H_2_O	Ammonium hydrogen carbonate 0.5 M

### 2.3. Processing, Sintering and Characterization

Simultaneous DTA-TG analyses (Netzsch STA409, Germany) were performed on powdered samples of about 150 mg, up to 1300 °C in static air, with a heating rate of 10 °C/min. The dried powders were calcined in the range 900–1500 °C, for 30 min, and their phase evolution was followed by X-Ray Diffraction (XRD, Philips PW1710, Eindhoren, The Netherlands).

Based on these analyses, the powders were pre-treated at 1000 °C for 30 min, in air, with a heating rate of 10 °C/min.

Green bodies were prepared by slip casting, which requires the preparation of stable suspensions with suitable solid loadings. For this reason, aqueous suspensions of the pre-treated powders were dispersed by ultrasonication (US). In order to increase the powder dispersibility and to yield stable suspensions, 3 wt% (as respect to the powder weight) of a commercial dispersant (Duramax D-3005, Rohm and Haas, Philadelphia, PA, USA) was added. The evolution of the particle size distribution within the dispersion time was determined by laser-granulometry (Fritsch Analysette 22 Compact, Germany).

The slurries (at 65 wt% solid loading) were cast into porous α-alumina molds and drying was performed in a humidity-controlled chamber for about 1 week.

Powders sinterability was investigated by absolute dilatometry (Netzsch 402E, Germany), under non-isothermal conditions, up to 1500 °C for 3 h (heating and cooling rate of 5 °C/min). The final densities were determined by Archimedes’s method and referred to YAG theoretical density (TD) of 4.55 g/cm^3^.

The fired microstructures were submitted to FESEM characterization (Hitachi S4000, High-Technologies Co., Tokyo, Japan), performed on polished and thermally etched surfaces. The grain size was determined by image analysis, carried out on several micrographs acquired by FESEM and using the Scandium Soft imaging system software.

## 3. Results and Discussion

### 3.1. Role of the AHC Precipitant Concentration

**Figure 1:**
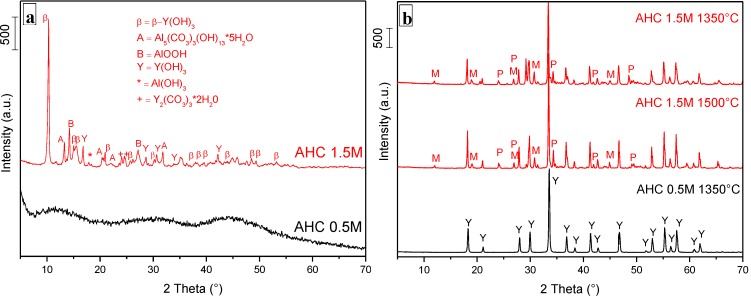
XRD patterns of YAG_cl_-AHC1.5 and YAG_cl_-AHC0.5 as (**a**) dried and (**b**) calcined at several temperatures (Y = YAG; P = perovskite YAlO_3_, M = monoclinic Y_4_Al_2_O_9_).

In Figure 1a, the XRD patterns of as-dried YAG_cl_-AHC0.5 and YAG_cl_-AHC1.5 are compared.

YAG_cl_-AHC0.5 showed a completely amorphous XRD diffraction pattern. As a comparison, the products derived from the separate precipitation of yttrium chloride and aluminum chlorides in AHC 0.5 M were also analyzed. By XRD (not shown), we observed an almost amorphous product in the case of the aluminum precipitate, whereas a crystalline product (precisely, a mixture of β-Y(OH)_3_, JCPDF No. 211448, and Y(OH)(CO_3_), JCPDF NO. 700278) was obtained by the precipitation of YCl_3_.6H_2_O. The lack of long-range order and the absence of crystalline species in such precipitate suggest a mixed distribution of Y and Al ions, thus preventing the formation of crystalline yttrium hydroxides and carbonates.

On the opposite, in the as-dried YAG_cl_-AHC1.5, we recognized carbonates and hydrated hydroxides, similarly to the previous results of Wang *et al.* [19]. More precisely, this precipitate was a mixture of crystalline phases, identified as Al_5_CO_3_(OH)_13_·5H_2_O (JCPDF NO. 420588), β-Y(OH)_3_ (JCPDF NO. 211448), boehmite AlOOH (JCPDF NO. 832384), gibbsite Al(OH)_3_ (JCPDF NO. 741775), Y_2_(CO_3_)_3_·2H_2_O (JCPDF NO. 24-1419) and Y(OH)_3_ (JCPDF NO. 731896). Some other unidentified XRD signals were also present. The composition of this precursor is the result of a competition between OH^−^ and the carbonate species, generated by the dissociation of ammonium bicarbonate in water, and subsequent chemical reaction with the metal cations [15].

The different phase composition in the as-dried products gave rise to a different phase development in the high-temperature calcined products. As shown in Figure 1b, YAG_cl_-AHC0.5 calcined at 1350 °C for 30 min produced pure YAG phase (JCPDF NO. 33-0040), whereas YAG_cl_-AHC1.5 treated at the same temperature yielded a mixture of several phases. In fact, beside the expected YAG, we observed the monoclinic Y_4_Al_2_O_9_ (YAM) (JCPDF NO. 211448), the perovskite YAlO_3_ (YAP) (JCPDF NO. 38-0222) and the α-Al_2_O_3_ phase (JCPDF NO. 10-0173). All these secondary phases were present also in the powders calcined at 1500 °C for 30 min, besides a more intense YAG phase. In addition, in both 1350 and 1500 °C-calcined powders, some unidentified XRD peaks were also detected.

These results highlight the key role of the precipitant concentration on the phase development and purity of the YAG phase. Some previous works have underlined the role of the AHC concentration on the formation of YAG [15,19]. For instance, Li *et al.* [15] used AHC at 1.5 M in order to avoid the precipitation of Al^3+^ ions as pseudo-boehmite (AlOOH) or ammonium dawsonite [NH_4_Al(OH)_2_CO_3_] which could occur at other AHC concentrations. Under these conditions, and contrary to our results, the authors obtained an amorphous precipitate and pure YAG at 900 °C. On the other side, our finding agrees with the work by Wang *et al.* [19], who obtained pure YAG at 1050 °C when using AHC at 0.5 M and an impure product, even after calcination at 1350 °C, when the AHC concentration was raised to 1.5 M. According to these authors, the YAG formation occurs by a solid reaction controlled by the diffusion of aluminum ions into the yttria particles. At high AHC concentrations, the size of yttria particles are at the micron scale and the alumina particles are in the form of clusters, due to the aggregation of smaller nuclei. Therefore, the powder homogeneity is relatively poor, and very high temperatures are necessary for the completion of phase transitions.

In any case, it should be underlined that all the previous results refer to YAG co-precipitated by aluminum and yttrium nitrates, contrary to the present results in which chlorides were used.

This result has a key importance toward the elaboration of transparent YAG material, since the presence of secondary phases dramatically reduces its optical properties [10]. Based on these results, the following experimentations were carried out only using AHC 0.5 M, whereas the effects of a higher concentration of AHC (1.5 M) will not be further discussed in this work.

### 3.2. Phase Evolution: Role of the Precipitant and the Precursors

YAG_cl_-AA, YAG_cl_-AHC0.5 as well as YAG_n_-AHC0.5 precipitates were completely amorphous, as observed by XRD (not shown).

**Figure 2:**
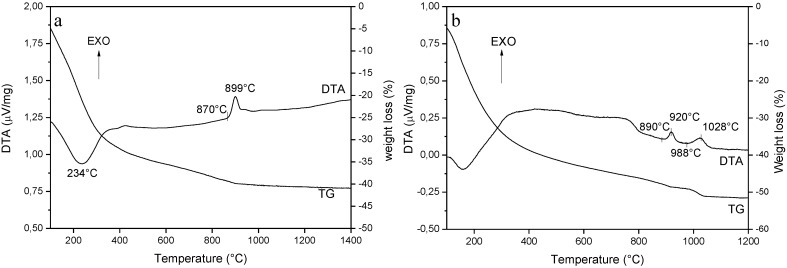
DTA-TG curves of (**a**) YAG_cl_-AA and (**b**) YAG_cl_-AHC0.5.

Figure 2 shows the DTA-TG curves of the as-dried YAG_cl_-AA (Figure 2a) and YAG_cl_-AHC0.5 (Figure 2b) powders.

YAG_cl_-AA underwent to an overall weight loss of about 40%. A significant mass loss occurred in the 100–400 °C range, associated to an important endothermic peak at about 230 °C, imputable to the progressive dehydration and stripping of OH^−^ groups from the amorphous precipitate [16]. In the higher-temperature DTA regime, we can observe an exothermic peak, whose onset was at about 970 °C, followed by a broad and slightly intense signal at about 950 °C, which can be ascribed to the crystallization of yttrium aluminates, as described below.

In the case of YAG_cl_-AHC0.5, we can observe a higher total weight loss (about 50%) as compared to YAG_cl_-AA, indicating a different phase composition. Major mass loss (about 85% of the total mass loss) occurred below 400 °C, imputable to dehydration and decomposition of partial carbonate. In the high-temperature DTA range, we can clearly observe two exothermal peaks, whose onset temperatures were at about 890 °C and 988 °C, respectively. As shown in the following, the first signal can be ascribed to the crystallization of the intermediate hexagonal-YAlO_3_, the second to its conversion into YAG at higher temperatures [16].

The DTA-TG curves of YAG_n_-AHC0.5 (not shown) are very similar to those of YAG_cl_-AHC0.5, with a total weight loss of about 45%, and two clear exothermic signals at about 915 °C and 1022 °C.

**Figure 3:**
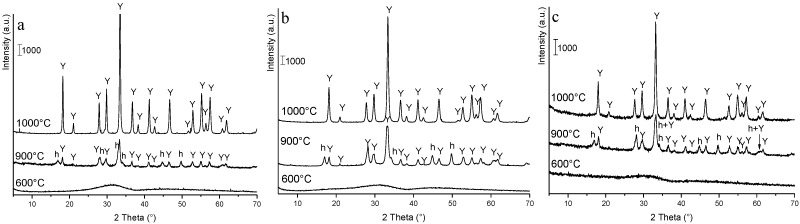
XRD patterns of (**a**) YAG_cl_-AA, (**b**) YAG_cl_-AHC0.5 and (**c**) YAG_n_-AHC0.5 calcined at 600 °C/1 h, 900 °C/30 min and 1000 °C/30 min. (In the figures: Y = YAG; h = hexagonal-YAlO_3_).

In Figure 3, the XRD patterns of YAG_cl_-AA (Figure 3a), YAG_cl_-AHC0.5 (Figure 3b) and YAG_n_-AHC0.5 (Figure 3c) calcined at 600, 900 and 1000 °C are shown. It is possible to observe a very similar phase evolution in the three products, supporting a minor role of both the precipitant and the precursors on the phase development.

The powders calcined at 600 °C were completely amorphous. Crystallization started at 900 °C, with the simultaneous formation of the hexagonal metastable YAlO_3_ and the stable YAG, for all three products. At 1000 °C, the hexagonal intermediate transformed to YAG, which was the only phase detected at this temperature. These data confirm the previous DTA results: the two exothermic signals observed in the three powders are due to the crystallization of h-YAlO_3_ (the former) and to its conversion to YAG (the latter).

In all cases, even after higher-temperatures treatments (up to 1600 °C, for 3 h), YAG was the only crystalline phase detected by XRD. This shows that co-precipitation is a suitable route to prepare pure YAG powders, and that both aqueous ammonia and ammonium hydrogen carbonate can be effectively used as precipitants. However, in the latter case, the precipitant concentration must be carefully selected, being the solution at 1.5 M unable to produce pure products.

In addition, when AHC 0.5 M is used, we have demonstrated that both chlorides and nitrates can be used as precursors, since they give rise to the same phase evolution and final phase.

In addition, this study allowed selecting the proper calcination pre-treatment of the powders, prior to forming and sintering. In fact, previous studies showed that the crystallization of YAG involves an abrupt shrinkage phenomenon, which can negatively affect the sinterability of the material [20]. For this reason, all the powders were calcined at 1000 °C for 30 min to yield a pure, well-crystallized YAG phase.

### 3.3. Processing and Sintering: Role of the Precipitant and the Precursors

YAG powders calcined at 1000 °C for 30 min were characterized by agglomerates of several tens of microns, independently of the precipitant and the precursors used. Thus, ultrasonication (US) was used to disperse the powders.

**Figure 4:**
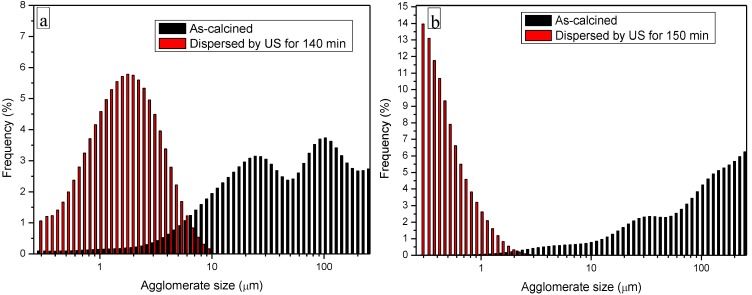
Frequency distribution (vol%) *vs.* agglomerate size of (**a**) YAG_cl_-AA and (**b**) YAG_cl_-AHC0.5 calcined at 1000 °C/30 min, before and after US dispersion.

In Figure 4, the frequency particle size distribution (by volume) of YAG_cl_-AA (Figure 4a) and YAG_cl_-AHC0.5 (Figure 4b) are compared. The as-calcined YAG_cl_-AA powder was characterized by a bimodal distribution, with an average agglomerate size of about 40 μm. The best dispersion conditions were reached after 140 min of US, when the powder reached a Gaussian distribution, with an average size of 1.6 μm. YAG_cl_-AHC0.5 was characterized by a larger, starting agglomerate size, of about 90 μm. However, such agglomerates were softer as compared to the YAG_cl_-AA ones, since after 150 min of US, a well-dispersed powder (average size of 0.4 μm) was obtained. In the case of YAG_n_-AHC0.5, the same behavior as YAG_cl_-AHC0.5 was observed, with a dispersed powder characterized by agglomerates of 0.5 μm as average value. However, here de-agglomeration was achieved after a longer dispersion time, since 220 min of US were necessary. Our results agree with the work of Li *et al.* [15], who suggested that the hard agglomerates obtained by precipitation in aqueous ammonia are due to hydrogen bonds, bridging adjacent particles with water. For the carbonate precursor, the possibility of hydrogen bond formation is significantly reduced, thus allowing softer precipitates.

The above-dispersed aqueous suspensions were employed to prepare slip cast green bodies, which were then pressureless sintered at 1500 °C/3 h or 1600 °C/3 h. The green and final density of the samples are collected in Table 2.

**Table 2:** Green and sintered density of the samples. In *Note*, the optical appearance of the materials.

Sample/sintering cycle	Green density g/cm^3^ (%TD)	Sintered density (%TD)	Grain size (μm)	*Note*
YAG_cl_-AA/1500 °C-3 h	2.23 (49.0)	96.3	0.61	Opaque
YAG_cl_-AA/1600 °C-3 h	2.31 (50.8)	96.3	n.d.	Opaque
YAG_cl_-AHC0.5/1500 °C-3 h	1.70 (37.4)	99.0	0.42	Translucent
YAG_cl_-AHC0.5/1600 °C-3 h	1.63 (35.8)	99.9	0.93	Translucent
YAG_n_-AHC0.5/1500 °C-3 h	1.45 (31.9)	99.4	0.33	Opaque

**Figure 5:**
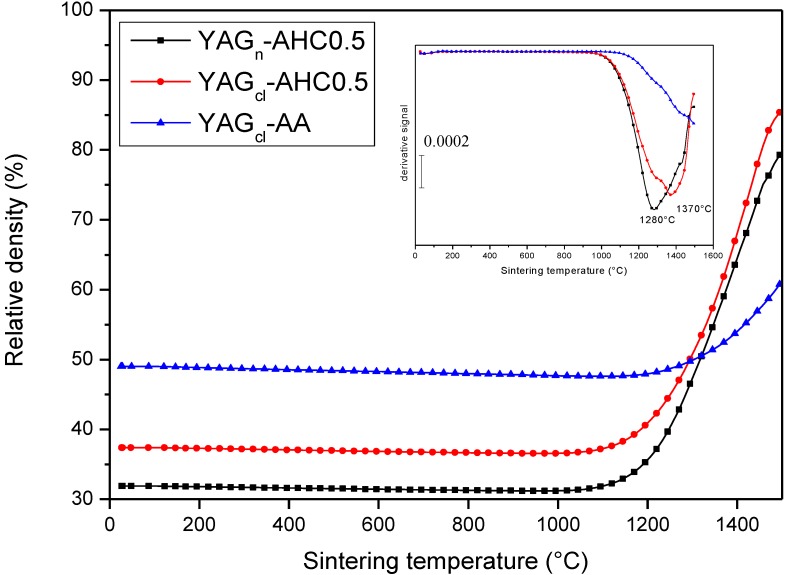
Relative density *vs.* sintering temperature of YAG_cl_-AA, YAG_cl_-AHC0.5 and YAG_n_-AHC0.5 samples. As an insert, the derivative signals of the same curves.

In Figure 5, the relative density of the three samples as a function of the sintering temperature is depicted. A clear role of the precipitant on the sintering behavior can be stated. We can observe in fact that the onset densification temperature is lower for the two AHC-precipitated products (about 1100 °C), as compared to the AA-precipitated one (about 1200 °C). In addition, YAG_cl_-AHC0.5 and YAG_n_-AHC0.5, during the heating step to 1500 °C, reached about 80% of the theoretical density. On the opposite, the density of YAG_cl_-AA increased in a very limited way during the heating step up to 1500 °C, moving from the starting value of about 50%, to about 60% TD. The three hours of isothermal steps were thus necessary for this sample to reach the high final density value reported in Table 2. In the inset of the same Figure, the corresponding derivative signals are reported, highlighting the different onset sintering temperatures of the materials. In addition, inflection points in the YAG_cl_-AHC0.5 and YAG_n_-AHC0.5 derivative curves can be observed, imputable to the temperature of maximum densification rate: such values were in the range 1300–1400 °C for the two AHC-derived materials. On the opposite, no inflection point, up to 1500 °C, could be detected in the derivative curve of YAG_cl_-AA sample.

After sintering, YAG_n_-AHC0.5 reached full densification, whereas YAG_cl_-AHC0.5 and YAG_cl_-AA achieved 99.0% and 96.3% TD, respectively. Therefore, these two last samples were submitted to a second sintering cycle, carried out at 1600 °C for 3 h. It allowed YAG_cl_-AHC0.5 to reach full densification, whereas any improvement in the final density was observed for YAG_cl_-AA.

A second difference between YAG_cl_-AHC0.5 and YAG_n_-AHC0.5 samples was observed in their optical properties. In fact, the former material, after sintering at both 1500 °C and 1600 °C, was translucent. On the opposite, even if fully dense, YAG_n_-AHC0.5 sintered at 1500 °C was completely opaque. As a possible explanation, we supposed that the longer US process, necessary to disperse the nitrates-derived YAG powder, could induce some pollution arising from the US probe itself (not detectable by XRD), affecting the optical properties of the material.

### 3.4. Microstructural Development: Role of the Precipitant and the Precursors

**Figure 6:**
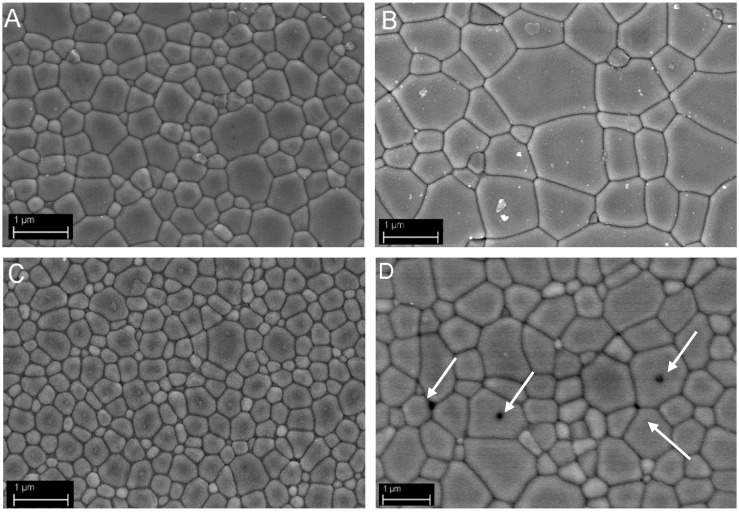
FESEM micrographs of YAG_cl_-AHC0.5 sintered at (**a**) 1500 °C/3 h and (**b**) 1600 °C/3 h and of (**c**) YAG_n_-AHC0.5 and (**d**) YAG_cl_-AA both sintered at 1500 °C/3 h.

In Figure 6, the FESEM micrographs of the sintered materials are collected. Their average grain sizes are given in Table 2. By comparing YAG_cl_-AHC0.5, YAG_n_-AHC0.5 and YAG_cl_-AA at the same sintering temperature, we can observe a different microstructural development. The materials were all homogeneous, with a narrow grain size distribution. In spite of this, YAG_n_-AHC0.5 presented the finer microstructure, with an average size of about 300 nm. On the opposite, YAG_cl_-AA showed the larger average grain size, of about 600 nm. In addition, some residual inter- and intra-granular pores (as pointed out by the arrows in Figure 6d) can be observed. The intermediate situation was presented by YAG_cl_-AHC0.5, showing a dense and fine microstructure with an average size of about 400 nm. However, after sintering at 1600 °C/3 h, a significant grain growth occurred in this material, giving rise to an average size of about 900 nm.

Once again, the key role of the precipitant can be stated. In fact, by using AHC, we obtained less agglomerated powders, characterized by higher sinterability, giving rise to denser and finer microstructures, as compared with the AA-derived material.

## 4. Conclusions

The fabrication of optically transparent polycrystalline YAG is still a challenge. It is imperative to achieve full density in the sintered materials and to avoid any secondary phase. In addition, a fine and homogeneous microstructure should be pursued in order to enhance both optical and mechanical properties. Keeping in mind these requirements, the aim of this work was to illustrate and discuss the role of different synthesis parameters during co-precipitation, which is one of the mostly used chemical processes to produce YAG powders.

In particular, we investigated the role of the precipitant, discussing aqueous ammonia (AA) *versus* ammonium hydrogen carbonate (AHC). The role of the AHC concentration was also investigated, by carrying out syntheses using 0.5 M and 1.5 M concentrations, respectively. Finally, when using AHC 0.5 M, the role of the YAG precursors was investigated, by comparing yttrium chlorides and nitrates.

Results highlight:

i) The key role of the AHC concentration. In fact, precipitation in AHC 0.5 M gave rise to an amorphous product and to pure YAG phase after calcination at 1000 °C, for 30 min. On the opposite, the use of AHC 1.5 M produced an almost well-crystallized precipitate, made by both hydroxide and carbonate species, suggesting a non-homogeneous distribution of the cations in the as-dried product. To strengthen this hypothesis, several secondary phases beside YAG were obtained, even after high temperature treatment (1500 °C, for 30 min);
ii) The key role of the precipitant. The use of AA induced a harder agglomeration in the powder calcined at 1000 °C, as compared to AHC. As a consequence, the powders derived by AHC precipitation showed better dispersibility and sinterability, giving rise to denser materials, with finer microstructures;
iii) When using AHC 0.5 M as a precipitant, a minor role of the YAG precursors was observed. In fact, both chlorides and nitrates gave rise to pure YAG powders, easily dispersed under ultrasonication and showing high sinterability;
iv) Finally, this work demonstrates that, when properly carried out, co-precipitation is an effective route to prepare YAG powders having the proper characteristics toward transparent ceramics. By using our best synthesis conditions (AHC at 0.5 M, by using both chlorides and nitrates as precursors), highly dense materials (99.0%–99.9%), with an ultra-fine microstructure (YAG average size of 300–400 nm) and showing translucent properties (in the case of chloride precursors) were successfully prepared.
